# A comparison between two rTMS protocols as augmentation strategies in patients with treatment-resistant depression

**DOI:** 10.1192/j.eurpsy.2023.311

**Published:** 2023-07-19

**Authors:** L. Larini, M. Castiglioni, E. Piccoli, C. Scarpa, M. Renne, S. Torriero, M. Bosi, B. Benatti, A. Varinelli, M. Vismara, B. Dell’Osso

**Affiliations:** 1 Psychiatry 2 Unit, Luigi Sacco Hospital; 2 “Aldo Ravelli” Center for Nanotechnology and Neurostimulation, University of Milan, Milan, Italy; 3 Department of Psychiatry and Behavioral Sciences, Stanford University, Stanford, United States

## Abstract

**Introduction:**

Repetitive transcranial magnetic stimulation (rTMS) is an evidence-based treatment and rTMS protocols have been included in international guidelines for patients with treatment-resistant depression (TRD). The daily administration of standard rTMS protocols, typically over several weeks, could be a limiting factor (e.g., time off from work, commuting issues). To intensify the antidepressant response and to reduce the number of stimulation days, it has been proposed that increasing the number of rTMS sessions performed per day could be more effective and help to reduce the burden for patients and clinicians. Although there is much interest in accelerated TMS protocols, little is known about their efficacy and tolerability, and the literature on the topic is still scarce.

**Objectives:**

To compare the efficacy and tolerability of two rTMS protocols (standard vs. accelerated) as augmentative strategies in patients with TRD.

**Methods:**

In the present ongoing, open-label, trial 14 patients meeting DSM-5 criteria for major depressive episode (either unipolar or bipolar), classified as partial responders or non-responders to adequate pharmacological treatment, were randomized to receive either standard (one session per day, five days a week, for four weeks; n= 7) or accelerated (two sessions per day, five days a week, for two weeks; n=6) rTMS treatment protocols. In both cases, rTMS was performed on the left dorsolateral prefrontal cortex, high frequency (10 Hz) at 120% of the motor threshold, 3000 pulses per sessions. Primary outcome measures included HAM-D, MADRS, and CGI-S scores at baseline (T0), at the end of rTMS treatment (T1), and after 1 month (T2), as well as tolerability based on adverse effects. Paired Samples *t*-Test for continuous variables was used to compare psychometric scales at each timepoint, while *t*-Test was used to compare differences between the two groups.

**Results:**

With respect to total sample, in terms of primary outcome measures a significant reduction of HAM-D, MADRS and CGI-S total scores between T0 and T1 (t: 3.01, p<0.05; t: 1.692, p<0.5; t:3.207, p<0.05 respectively), T1 and T2 (t: 3.264, p<0.05: t:2.669, p<0.05; t:.085, p=0.437 respectively) and T0 and T2 (t:5.669, p<0.05; t=4.711, p<0.05; t:2.551, p<0.05 respectively) was found. No significant differences in terms of efficacy were found between the two groups. One patient dropped-out for reasons not related to rTMS treatment. Mild and transient headache during the stimulation was the only side effect reported (4 patients).

**Image:**

**Figure:**
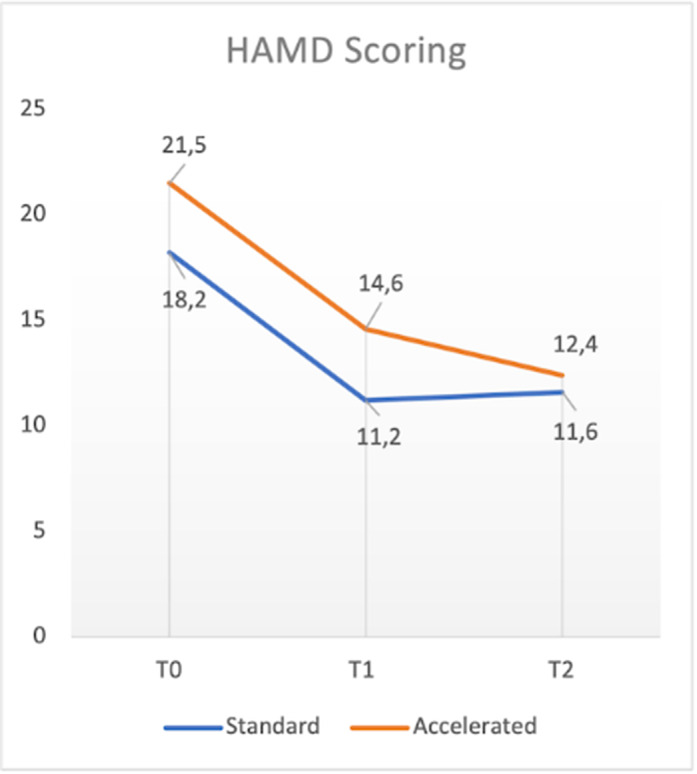

**Conclusions:**

Consistently with previous literature studies, our preliminary results supported the evidence of comparable efficacy and tolerability between accelerated and standard rTMS protocols. In the future, larger, blinded, and controlled trials might support these conclusions and further address treatment parameters of novel accelerated rTMS protocols.

**Disclosure of Interest:**

None Declared

